# Autonomic thermoregulatory responses and subjective thermal perceptions upon the initiation of thermal behavior among resting humans in hot and humid environment

**DOI:** 10.1186/s40101-022-00308-x

**Published:** 2022-10-10

**Authors:** Keneth B. Sedilla, Takafumi Maeda

**Affiliations:** 1 Department of Design (Human Science International Course), Graduate School of Design, Kyushu University, 4-9-1 Shiobaru, Minami-ku, 815-8540 Fukuoka, Japan; 2 Department of Human Life Design and Science, Faculty of Design, Kyushu University, 4-9-1 Shiobaru, Minami-ku, 815-8540 Fukuoka, Japan; 3 Physiological Anthropology Research Center, Faculty of Design, Kyushu University, 4-9-1 Shiobaru, Minami-ku, 815-8540 Fukuoka, Japan

**Keywords:** Behavioral thermoregulation, Autonomic responses, Subjective responses, Hot and humid environment

## Abstract

**Background:**

While thermoregulatory behavior is critical for maintaining homeostasis, our knowledge of behavioral thermoeffectors in humid heat is limited compared to the control of autonomic thermoeffectors. The predictions that the frequency and duration of intensified humid heat events are expected to increase in the coming years underline this shortcoming. Therefore, this study aims to elucidate the activation of autonomic thermoregulatory responses and subjective thermal perceptions upon deciding to initiate thermal behavior in a hot and humid environment.

**Methods:**

Ten young male adults participated in an experimental trial where local cooling was permitted at any time during the behavioral assessment during passive exposure to humid heat. The air temperature and relative humidity were kept at 33$^{\circ }$C and 80$\%$, respectively. Skin temperatures, core body temperature (T$_{\text {core}}$), and skin blood flow (forearm, upper arm, and upper back) were obtained 120 s preceding thermal behavior. Local sweat rate (forearm and upper arm) and subjective thermal perceptions (neck and whole-body) upon thermal behavior initiation were also recorded.

**Results:**

Mean skin temperature (${\overline{\mathrm {T}}}_{\text {sk}}$) and T$_{\text {core}}$ increased prior to thermal behavior initiation (*p* $=$ 0.002; *p* $=$ 0.001). An increase in mean body temperature (${\overline{\mathrm {T}}}_{\text {body}}$) was also observed (*p* < 0.001). However, the initiation of thermal behavior is not preceded by an increase in skin blood flow (*p* $\ge$ 0.154) and local sweat rate (*p* $\ge$ 0.169). An increase in thermal discomfort and skin wetness perception was observed (*p* $\le$ 0.048; *p* $\le$ 0.048), while thermal sensation did not differ from the baseline (*p* $\ge$ 0.357).

**Conclusion:**

These findings suggest that when given the opportunity to behaviorally thermoregulate in a hot and humid environment, changes in skin blood flow and sweat rate are not required for thermal behavior to be initiated in resting humans. Moreover, an increase in ${\overline{\mathrm {T}}}_{\text {sk}}$ and T$_{\text {core}}$, which appears to cause an increase in thermal discomfort, precedes thermal behavior. In addition, an increase in ${\overline{\mathrm {T}}}_{\text {body}}$ leading up to thermal behavior initiation was observed, suggesting that changes in ${\overline{\mathrm {T}}}_{\text {body}}$ rather than ${\overline{\mathrm {T}}}_{\text {sk}}$ and T$_{\text {core}}$ alone mediate thermal behavior in humid heat. Collectively, the results of this study appear to support the hypothesis that the temporal recruitment of autonomic thermoeffectors follows an orderly manner based on their physiological cost.

## Background

When humans are subjected to heat stress, the temperature gradient between the skin and the environment is reduced, causing a decrease in heat dissipation rate [1]. As a result, the body becomes heavily dependent on evaporative cooling via sweating to facilitate heat loss [2]. However, evaporative heat loss decreases when humidity rises due to a reduced water vapor pressure gradient between the ambient air and the skin’s surface [1]. Such hot and humid condition significantly affects thermal comfort [3] and elevates skin temperature (T$_{\mathrm {sk}}$) and core body temperature (T$_{\text {core}}$) [4].

Thermal behavior has long been recognized as our first line of defense against uncompensable heat stress [5, 6]. Behavioral thermoeffectors have been postulated to alleviate the autonomic strain by preventing the rise in T$_{\text {core}}$ and thereby minimizing the requirement to activate cutaneous vasodilation and sweating [7]. While it is critical for homeostasis, our understanding of behavioral thermoregulation is limited compared to the substantial work on autonomic thermoregulation.

Recent studies on behavioral thermoregulation in resting humans have utilized paradigms that permit thermal behavior while continuously measuring autonomic responses along with subjective thermal perceptions upon initiating thermal behavior [5, 8–13]. Schlader et al. [5, 8, 9, 11, 13] conducted several studies using the shuttle-box thermoregulatory model that permits passive movement between a cold room (air temperature [T$_{\text {air}}$]: 7–17 $^{\circ }$C, relative humidity [RH]: 31–50%) and a warm room (T$_{\text {air}}$: 40–45$^{\circ }$C, RH: 10–47%), when they feel “too cool” or “too warm”. They found that neither sweating nor shivering is required to initiate thermal behavior [8]. It has also been reported that thermal behavior is preceded by small changes in skin blood flow (SkBF) and occurs prior to a substantial increase in SkBF and sweating during heat exposure or an increase in metabolic heat production during cold exposure [14]. Furthermore, Schlader et al. [5] suggest that thermal behavior is elicited by subjective thermal perceptions caused by changes in mean T$_{\mathrm {sk}}$ (${\overline{\mathrm {T}}}_{\text {sk}}$) and T$_{\text {core}}$. These findings, taken together, suggest that autonomic thermoeffectors are recruited in a systematic and coordinated manner relative to their physiological costs [10].

While the previous literature has advanced our understanding of human behavioral thermoregulation, its mechanisms and modulators under extreme thermal environments remain unknown. Furthermore, these studies have utilized experimental paradigms by which thermal behavior in resting humans is examined in a dry heat environment. To our knowledge, the subjective thermal perceptions and the temporal recruitment of autonomic thermoeffectors preceding thermal behavior in a hot and humid environment have not been formally examined. The predictions that the frequency and duration of intensified humid heat events are expected to increase in the coming years [15] underline this shortcoming.

Therefore, this study aims to provide insights into human behavioral thermoregulation in a hot and humid environment. Moreover, this study aimed to test whether the systematic recruitment of autonomic thermoeffectors holds true in hot and humid conditions. Since evaporative heat loss is impeded in humid heat, ${\overline{\mathrm {T}}}_{\text {sk}}$ and T$_{\text {core}}$ are likely to be substantially elevated. Thus, we hypothesized that changes in ${\overline{\mathrm {T}}}_{\text {sk}}$ and T$_{\text {core}}$ precede thermoregulatory behavior. However, we expect that there will be no changes in SkBF and local sweat rate (LSR) leading up to and upon thermal behavior initiation.

## Methods

### Subjects

Ten young male adults participated in this study. The subjects’ characteristics were as follows: age, 23 ± 2 years; height, 171.5 ± 5.1 cm; weight, 64.9 ± 7.8 kg; body surface area, 1.76 ± 0.11 m$^{2}$; percent body fat, 14.07 ± 4.51$\%$. All subjects were physically or recreationally active and not taking any medications. The Montreal Cognitive Assessment (MoCA) [16] was used to ensure that the subjects did not have any mild cognitive impairment (MoCA score: 29 ± 1). The subjects were free from any known cardiovascular, respiratory, or neurological diseases. All the subjects abstained from alcoholic drinks and vigorous exercise for 24 h and did not consume any food or caffeine for at least 2 h before the experiment. Each subject was fully informed of the experimental procedures and possible risks before obtaining written informed consent. Experimental testing was conducted from winter to spring in Fukuoka, Japan (the average outdoor temperature on the day and time of the experimental trials was 16 ± 4$^{\circ }$C). Within the month leading up to the experimental testing, none of the participants had performed any training in particularly hot environments. This study was approved by the Ethics Committee of the Graduate School of Design, Kyushu University (approval number 394).

### Experimental design and procedures

Subjects arrived at the laboratory euhydrated (urine specific gravity: 1.05 ± 0.03) and wore short-sleeve T-shirts and shorts (0.13 clo). Following instrumentation in the adjacent room with T$_{\text {air}}$ at 25 $^{\circ }$C and 50% RH, subjects were transferred to the climatic chamber and assumed a seated position on a standard upright cycle ergometer with custom-made footstools and armrests. Throughout the experiment, the climatic chamber’s T$_{\text {air}}$ and RH were fixed at 33 $^{\circ }$C and 80%, respectively.

The experimental trial lasted 60 min and was separated into two phases: baseline measurement (which occurred within the first 5 minutes of the experiment) and behavioral assessment. At any time during the behavioral assessment phase, subjects were allowed to use a portable cooler (IPC-221N, Iris Ohyama Inc., Japan) with a custom-built tubing system that delivers cool air ($\sim$ 6 m s$^{-1}$) to the dorsal part of their neck. They were specifically instructed to use the portable cooler to keep their neck at a thermally comfortable temperature throughout the experiment. This local-cooling behavioral paradigm, in which cooling is permitted to maintain local thermal comfort [14], was adopted and modified from those of Schlader et al. [10]. In our study, the initiation of thermal behavior was defined as the decision to use and/or adjust the temperature of the portable cooler. All subjects were allowed to watch nature and ecology documentaries (i.e., “Our Planet”) throughout the experiment.

### Measurements

T$_{\text {core}}$ and all T$_{\mathrm {sk}}$ (including the dorsal neck temperature [T$_{\text {neck}}$]) were recorded every 1 s using a data logger (LT-8A, Gram Corporation, Japan; precision: ± 0.01$^{\circ }$C). In this study, aural temperature served as an indicator of T$_{\text {core}}$ and was measured with earplug-type thermistor (Nikkiso-Thermco. Ltd., Japan; maximum precision: ± 0.01 $^{\circ }$C within 30–40 $^{\circ }$C temperature range). ${\overline{\mathrm {T}}}_{\text {sk}}$ was obtained as the weighted average of ten skin thermistors (LT-ST08-12, Gram Corporation, Japan; precision: ± 0.01 $^{\circ }$C) attached to the following locations: forehead, upper back, chest, upper arm, forearm, hand, abdomen, thigh, lower leg, and instep [17], while mean body temperature (${\overline{\mathrm {T}}}_{\text {body}}$) was calculated as $0.9 \times \mathrm {T}_{\text {core}} + 0.1 \times {\overline{\mathrm {T}}}_{\text {sk}}$ [9].

SkBF was measured every 0.01 s (then averaged to 1 s) with a laser Doppler flowmeter using a contact-type disk probe (Omegaflo FLO-C1, Omegawave Co., Ltd, Japan; time constant: 1 s) at three locations: on the dorsal surface of the proximal third of the left forearm (SkBF$_{\mathrm {fa}}$), on the left upper arm (SkBF$_{\mathrm {ua}}$), and on the upper back (SkBF$_{\mathrm {ub}}$).

LSR was obtained by securing a plastic capsule infused with dry silica gel on the dorsal surface of the right forearm (LSR$_{\mathrm {fa}}$) and upper arm (LSR$_{\mathrm {ua}}$) with a doughnut-shaped double-sided medical tape (Nihon Kohden Corp., Japan). The plastic capsule has a circular opening at the bottom, covering 1 cm$^{2}$ of the skin. The LSR was calculated by taking the difference in mass of the plastic capsule (with silica gel) between the post- and pre-application (measured in triplicate), dividing by the surface area covered by the plastic capsule and the duration of application, yielding values in mg min$^{-1}$ cm$^{-2}$. Throughout the experimental trial, LSR$_{\mathrm {fa}}$ and LSR$_{\mathrm {ua}}$ were measured at a 5-min interval and linear interpolation was used to determine the LSRs at the time when thermal behavior was first initiated.

The participants used subjective scales [18, 19] to rate their thermal sensation (TS), skin wetness perception (SW), and thermal comfort (TC) on their dorsal neck and across their whole body at the time when thermal behavior was first initiated. Subjective thermal perceptions were also recorded every 5 min throughout the experimental trial.

### Data analysis

In this study, our data analysis focused exclusively on the subject’s initial thermal behavior, which occurred when they turned on the portable cooler for the first time. The analysis of the subsequent behavioral responses was not included in this study.

Temperature and SkBF data were analyzed at baseline (5-min average) and 120, 90, 60, and 30 s (all 30 s averages) immediately before the initiation of thermal behavior. These data were also reported as a percent change (%$\Delta$) from 120 s preceding thermal behavior, which enabled the identification of changes in these physiological measurements (adopted from Schlader et al. [8]). LSR data and subjective thermal perceptions were analyzed at baseline and at the time when the thermal behavior was first initiated. All temperature and SkBF data were analyzed using one-way repeated measures ANOVA. Data were assessed for approximation to a normal distribution and sphericity. Where appropriate, post hoc Bonferroni adjusted pair-wise comparisons were made. A paired *t*-test was used to analyze all LSR data, while related Wilcoxon signed-rank test was used to analyze subjective thermal perceptions. Data were analyzed using SPSS v. 28.0. A priori statistical significance was set at *p* $\le$ 0.05, and all data were presented as mean ± SD.

## Results

Two subjects did not use the portable cooler throughout the experimental trial. The average time before the initiation of thermal behavior was 12.0 ± 10.0 min (n = 8), and the individual latency before the initiation of thermal behavior was 6.5, 6.1, 35.3, 6.4, 8.1, 6.1, 11.9, and 15.5 min, respectively.

### Body temperatures

**Fig. 1:**
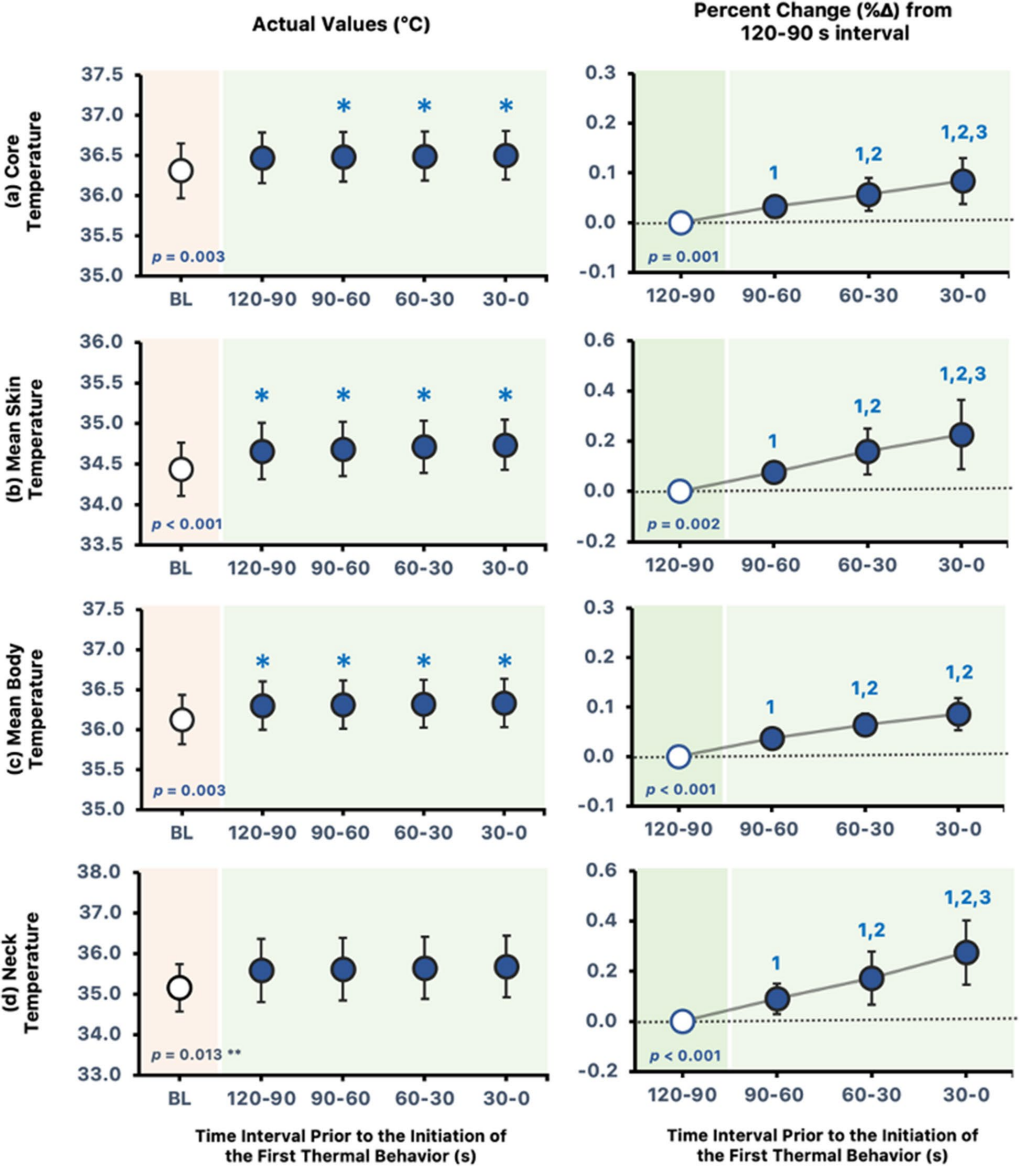
**a** T$$_{\text {core}}$$, **b** $${\overline{\mathrm {T}}}_{\text {sk}}$$, **c** $${\overline{\mathrm {T}}}_{\text {body}}$$, and **d** T$$_{\text {neck}}$$ at baseline and 120, 90, 60, and 30 s immediately before thermal behavior (left panels) and the %$$\Delta$$ from the 120–90-s interval preceding thermal behavior (right panels). All values are reported as means ± SD, *n* = 8. $$^{*}$$Different from baseline (*p* $$\le$$ 0.05). $$^{1}$$Different from 120–90-s interval (*p* $$\le$$ 0.024). $$^{2}$$Different from 90–60-s interval (*p* $$\le$$ 0.02). $$^{3}$$Different from 60–30-s interval (*p* $$\le$$ 0.029). $$^{**}$$The one-way repeated measures ANOVA is significant; however, post hoc analysis indicates that all 30-s intervals prior to thermal behavior initiation are not different from the baseline (*p* $$\ge$$ 0.089). The *p*-values for one-way repeated measures ANOVA are noted

At all time intervals preceding thermal behavior, T$_{\text {core}}$ was higher than baseline (*p* $\le$ 0.042; Fig. 1a) except for the 120–90-s interval (*p* $=$ 0.059), whereas the %$\Delta$ from 120 to 90 s before thermal behavior in T$_{\text {core}}$ was increasing (*p* = 0.001). Likewise, both ${\overline{\mathrm {T}}}_{\text {sk}}$ and ${\overline{\mathrm {T}}}_{\text {body}}$ were higher than baseline preceding thermal behavior (*p* $\le$ 0.05; Fig. 1b and c) and elicited an increasing trend in the %$\Delta$ from 120 to 90 s immediately before thermal behavior (*p* $\le$ 0.002). T$_{\text {neck}}$ did not differ between baseline (*p* $\ge$ 0.089; Fig. 1d) but was increasing in the %$\Delta$ from 120 to 90 s immediately before thermal behavior (*p* < 0.001).

### Skin blood flow

**Fig. 2:**
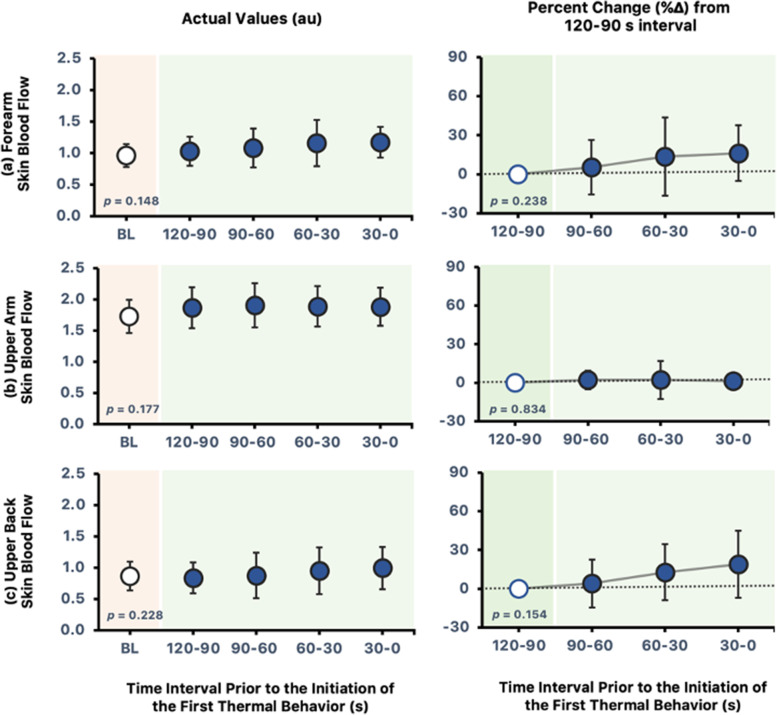
**a** SkBF$_{\mathrm {fa}}$, **b** SkBF$_{\mathrm {ua}}$, and **c** SkBF$_{\mathrm {ub}}$ at baseline and 120, 90, 60, and 30 s immediately before thermal behavior (left panels) and the %$\Delta$ from the 120–90-s interval preceding thermal behavior (right panels). All values are reported as means ± SD, *n* = 8. The *p*-values for one-way repeated measures ANOVA are noted

SkBF$_{\mathrm {fa}}$ did not differ from baseline at all time intervals preceding thermal behavior (*p* = 0.148; Fig. 2a). The %$\Delta$ from 120 s preceding initiation of thermal behavior in SkBF$_{\mathrm {fa}}$ did not change (*p* = 0.238). Similarly, both SkBF$_{\mathrm {ua}}$ and SkBF$_{\mathrm {ub}}$ were not different from baseline (*p* $\ge$ 0.177; Fig. 2b and c), and there were no changes in the %$\Delta$ from 120 to 90 s immediately before thermal behavior (*p* $\ge$ 0.154).

### Local sweat rate

**Fig. 3:**
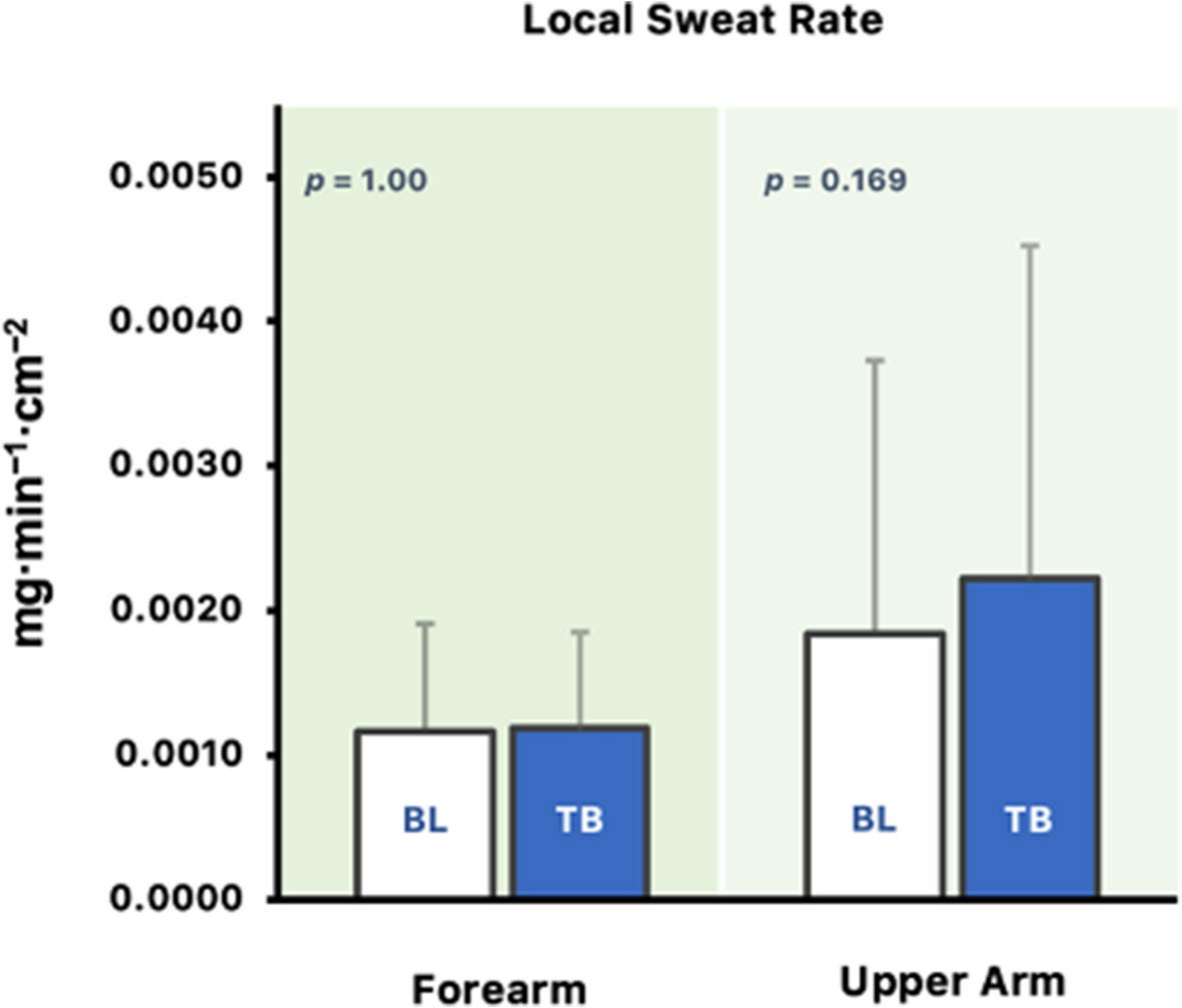
Paired *t*-test results for LSR$_{\mathrm {fa}}$ and LSR$_{\mathrm {ua}}$ at baseline (BL) and at the time when thermal behavior was initiated (TB). All values are reported as means ± SD, *n* = 8. The *p*-values for paired *t*-tests are noted

LSR$_{\mathrm {fa}}$ at the time when thermal behavior was initiated was not different from baseline (*p* = 1.00; Fig. 3). Similarly, the same results were observed for LSR$_{\mathrm {ua}}$ (*p* = 0.169).

### Subjective thermal perceptions

**Fig. 4:**
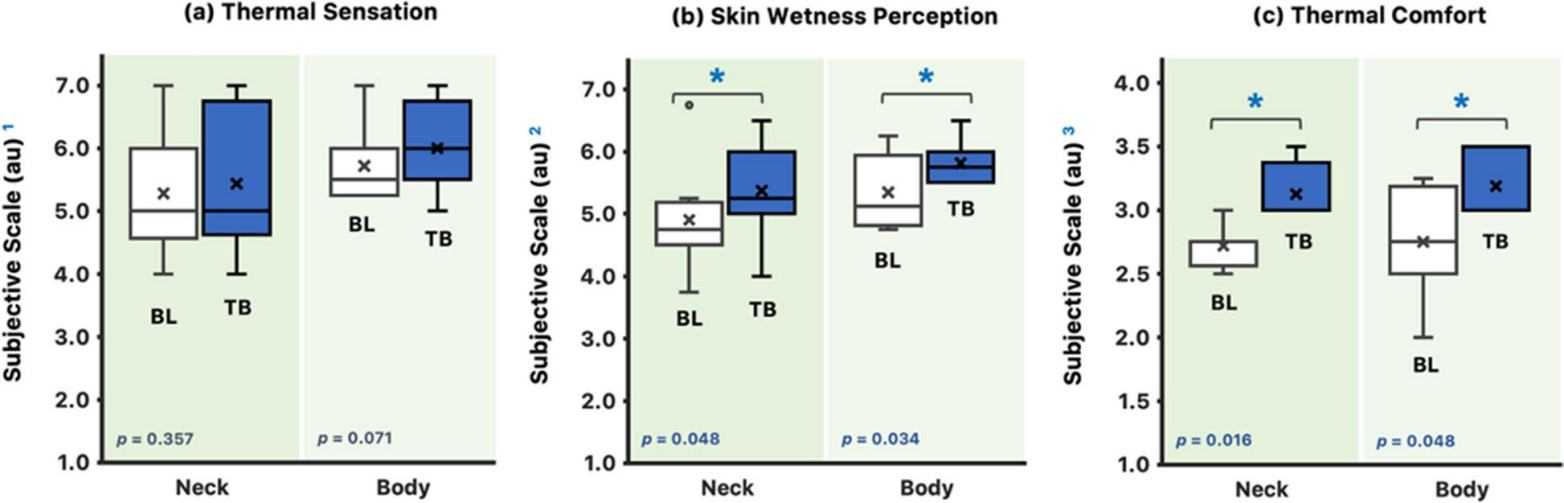
Boxplot representation and related Wilcoxon signed-rank test results for **a** TS, **b** SW, and **c** TC at baseline (BL) and at the time when thermal behavior was initiated (TB). $^{1}$TS subjective scale [18] (to the nearest 0.5 units; 1 = cold, 2 = cool, 3 = slightly cool, 4 = neutral, 5 = slightly warm, 6 = warm, 7 = hot). $^{2}$SW subjective scale [19] (to the nearest 0.5 units; 1 = very dry, 2 = dry, 3 = slightly dry, 4 = neutral, 5 = slightly wet, 6 = wet, 7 = very wet). $^{3}$TC subjective scale [18] (to the nearest 0.5 units; 1 = comfortable, 2 = slightly comfortable, 3 = uncomfortable, 4 = very uncomfortable). $^{*}$The median of differences is significantly different (*p* $\le$ 0.048). The *p*-values for related Wilcoxon signed-rank test are noted (*n *= 8)

Subjects felt “warm” at the time when thermal behavior was initiated, and it was not different from baseline (TS$_{\text {body}}$: *p* = 0.071; Fig. 4a). Furthermore, subjects felt “slightly warm” in their neck region at thermal behavior initiation, and it did not differ from baseline (TS$_{\text {neck}}$: *p* = 0.357). Subjects perceived their neck region (SW$_{\text {neck}}$) and their whole body (SW$_{\text {body}}$) “slightly wet” at the time when thermal behavior was initiated (*p* $\le$ 0.048; Fig. 4b), and both differed from the baseline. For TC$_{\text {body}}$ and TC$_{\text {neck}}$, subjects felt “slightly uncomfortable” at baseline but felt “uncomfortable” at thermal behavior initiation. Both TC$_{\mathrm {body}}$ and TC$_{\text {neck}}$ were different from baseline (*p* $\le$ 0.048; Fig. 4c).

## Discussion

In support of our hypothesis, this study demonstrates an increase in ${\overline{\mathrm {T}}}_{\text {sk}}$ and T$_{\text {core}}$ upon the initiation of thermal behavior. Likewise, an increase in ${\overline{\mathrm {T}}}_{\text {body}}$ were observed preceding initial thermal behavior. However, it is not preceded by an increase in SkBF and LSR, indicating that changes in skin blood flow and sweating are not required for the initiation of thermal behavior in a hot and humid environment. Furthermore, changes in SW and TC were observed upon thermal behavior initiation.

### Autonomic and subjective responses upon the initiation of thermal behavior

In resting humans, Schlader et al. [8] have reported that behavioral responses are primarily driven by signals arising from changes in ${\overline{\mathrm {T}}}_{\text {sk}}$ and not in T$_{\text {core}}$. The present study, however, found that both ${\overline{\mathrm {T}}}_{\text {sk}}$ and T$_{\text {core}}$ increased prior to the initiation of thermal behavior. In addition, the results showed that T$_{\text {core}}$ and ${\overline{\mathrm {T}}}_{\text {sk}}$ were higher than baseline at nearly all time points leading up to thermal behavior initiation. Thus, it is possible that both the absolute values and the changes in T$_{\text {core}}$ and ${\overline{\mathrm {T}}}_{\text {sk}}$ influence the decision to thermoregulate behaviorally. We were not able to uncover the related mechanisms of these findings in our study design. Thus, further research is required to determine whether the absolute temperature or the rate of increase in temperature influences thermal behavior or a combination of both. We have also observed an increase in ${\overline{\mathrm {T}}}_{\text {body}}$ immediately before thermal behavior. These findings suggest that in hot and humid environments, both ${\overline{\mathrm {T}}}_{\text {sk}}$ and T$_{\text {core}}$ appear to be important thermal inputs in signaling a subjective, conscious increase in thermal discomfort, which subsequently induces thermal behavior. Furthermore, our results corroborate prior findings [20] that changes in ${\overline{\mathrm {T}}}_{\text {body}}$, rather than ${\overline{\mathrm {T}}}_{\text {sk}}$ alone, mediate thermal behavior initiation. These results are not surprising given the previously demonstrated significant correlation between ${\overline{\mathrm {T}}}_{\text {sk}}$ and thermal discomfort [5].

Thermal behavior has also been reported to be preceded by slight changes in SkBF and occurs before the significant increase in SkBF and sweating during heat exposure [8]. Similarly, this study demonstrated the same results where marginal increase in SkBF precede thermal behavior activation. Moreover, no changes were observed in LSRs at the point when thermal behavior was initiated. From these results, it can be postulated that changes in skin blood flow and sweat rate are not required to initiate thermal behavior in hot and humid environments.

Humidity is a significant limiting factor in the evaporation of perspiration in humid heat [21]. It has been proposed that perceived changes in ambient humidity and absolute skin wetness influence thermal comfort [22]. In this study, some of the generated sweat drips from the body due to the high level of humidity. The subjects perceived “slight skin wetness” upon thermal behavior initiation. Thus, we speculate that the accumulated non-evaporated sweat on the skin exacerbates thermal discomfort. It is possible that in the current paradigm, the autonomic thermoeffector end-organ responses (increase in body temperatures and skin wetness) are sensed via known afferent signaling pathways and interpreted as thermal discomfort, which ultimately stimulates behavior [23]. Unfortunately, we were not able to measure the actual skin wetness during the experimental trial, so we cannot speculate the degree of its potential role in thermal behavior initiation. Nevertheless, such a relationship appears to be probable.

### Recruitment of thermoeffectors

Recent studies have found that thermal behavior reduces the need for autonomic thermoeffector activation, enabling body temperature regulation with minimal impact on the regulation of other biological systems [10, 14]. Schlader et al. [14] hypothesized that autonomic thermoeffector activation is sensed and recruited in an orderly manner to stimulate thermal behavior and that autonomic thermoeffectors that do not consume many physiological resources are activated before the more resource-consuming thermoeffectors. Collectively, our findings that the initiation of thermal behavior is preceded by an increase in ${\overline{\mathrm {T}}}_{\text {sk}}$ and T$_{\text {core}}$ and that changes in SkBF and LSR are not required in thermal behavior activation appear to support the orderly recruitment of autonomic thermoeffectors.

### Considerations and perspectives

In this study, several methodological considerations warrant attention. First, our findings are constrained by the methodology employed herein. For instance, throughout the experimental trial, the climatic chamber’s T$_{\text {air}}$ and RH were fixed at 33 $^{\circ }$C and 80%, respectively. Thus, whether our results would have been the same if we had used different T$_{\text {air}}$ and RH in simulating hot and humid environments remains unknown. Moreover, skin blood flow measurements were localized to the forearm, upper arm, and upper back, while sweat rate was measured in the forearm and upper arm. During heat exposure, variations in skin blood flow and sweat rate are known to occur across the body. Therefore, it is unknown whether our findings can be extended to other body measurement sites, such as those with glabrous skin. Also, it is essential to note that local sweat rate data at the thermal behavior initiation were estimated using linear interpolation, so they might not have been entirely accurate. Furthermore, we did not control for the time of day of the experimental trials. Second, we did not perform an a priori power analysis in determining sample size. However, our primary measurement variables, such as T$_{\text {core}}$, ${\overline{\mathrm {T}}}_{\text {sk}}$, ${\overline{\mathrm {T}}}_{\text {body}}$, and T$_{\text {neck}}$, which show statistically significant changes leading up to the initiation of thermal behavior, all reached a statistical power of at least 96.78%. Lastly, the present study utilized only male subjects. Given that sex modulates autonomic and subjective responses, sex-related differences in behavioral thermoregulation appear likely. Therefore, formal comparisons between male and female subjects are warranted.

## Conclusion

This study demonstrates that when given the opportunity to behaviorally thermoregulate in a hot and humid environment, changes in skin blood flow and an increase in sweat rate are not required for thermal behavior to be initiated in resting humans. Furthermore, an increase in ${\overline{\mathrm {T}}}_{\text {sk}}$ and T$_{\text {core}}$, which appears to cause an increase in thermal discomfort, precedes the decision to thermoregulate behaviorally. In addition, an increase in ${\overline{\mathrm {T}}}_{\text {body}}$ leading up to thermal behavior initiation was observed, suggesting that changes in ${\overline{\mathrm {T}}}_{\text {body}}$ rather than ${\overline{\mathrm {T}}}_{\text {sk}}$ and T$_{\text {core}}$ alone mediate thermal behavior in humid heat. Altogether, these findings suggest that the orderly recruitment of autonomic thermoeffectors holds true in hot and humid conditions.

Nonetheless, to the best of our knowledge, this is the first study of its sort. Thus, this study can be considered a preliminary step in understanding the control of human behavioral thermoregulation in hot and humid environments. We believe that knowledge regarding the mechanisms and modulators of thermal behavior in humid heat is essential in light of forecasts that the frequency and duration of intense humid heat will rise in the coming years.

## Data Availability

All datasets used and/or analyzed in this study are available from the corresponding authors on reasonable request.

## Abbreviations

T_air_: Air temperature
RH: Relative humidity
T_sk_: Skin temperature
T_core_: Core body temperature
${\overline{\mathrm {T}}}_{\text {sk}}$: Mean skin temperature
${\overline{\mathrm {T}}}_{\text {body}}$: Mean body temperature
$$\mathrm {T}_{\text {neck}}$$: Neck temperature
SkBF$_{\mathrm {fa}}$: Forearm skin blood flow
SkBF$_{\mathrm {ua}}$: Upper arm skin blood flow
SkBF$_{\mathrm {ub}}$: Upper back skin blood flow
LSR$_{\mathrm {fa}}$: Forearm local sweat rate
LSR$_{\mathrm {ua}}$: Upper arm local sweat rate
TS$_{\text {body}}$: Whole-body thermal sensation
TS$_{\text {neck}}$: Neck thermal sensation
TC$_{\text {body}}$: Whole-body thermal comfort
TC$_{\text {neck}}$: Neck thermal comfort
SW$_{\text {body}}$: Whole-body skin wetness perception
SW$_{\text {neck}}$: Neck skin wetness perception
%$$\Delta$$: Percent change
BL: Baseline
TB: Thermal behavior
